# A prospective randomized study comparing bipolar plasmakinetic transurethral resection of the prostate and monopolar transurethral resection of the prostate for the treatment of Benign Prostatic Hyperplasia: efficacy, sexual function, Quality of Life, and complications

**DOI:** 10.1590/S1677-5538.IBJU.2019.0766

**Published:** 2020-11-18

**Authors:** Hugo Otaola-Arca, Manuel Álvarez-Ardura, Roberto Molina-Escudero, Mario I. Fernández, Álvaro Páez-Borda

**Affiliations:** 1 Clínica Alemana Department of Urology Santiago Chile Department of Urology, Clínica Alemana, Santiago, Chile; 2 Hospital Universitario de Fuenlabrada Department of Urology Madrid Spain Department of Urology, Hospital Universitario de Fuenlabrada, Madrid, Spain; 3 Universidad Rey Juan Carlos International Doctoral School Madrid Spain International Doctoral School, Universidad Rey Juan Carlos, Madrid, Spain; 4 Clínica Alemena-Universidad del Desarrollo Faculty of Medicine Santiago Chile Faculty of Medicine, Clínica Alemena-Universidad del Desarrollo, Santiago, Chile

**Keywords:** Transurethral Resection of Prostate, Quality of Life, Prostatic Hyperplasia

## Abstract

**Objective:**

To generate high-quality data comparing the clinical efficacy and safety profile between monopolar transurethral resection of the prostate (M-TURP) and bipolar plasmakinetic resection of the prostate (PK-TURP) for benign prostatic hyperplasia (BPH).

**Materials and Methods:**

Prospective, randomized, single-blinded study conducted in a tertiary-care public institution (Dec/2014-Aug/2016). Inclusion criteria: prostate of <80g in patients with drug-refractory lower urinary tract symptoms (LUTS), complications derived from BPH, or both. Exclusion criteria: a history of pelvic surgery/radiotherapy, neurogenic bladder dysfunction or documented/suspected prostate carcinoma. Treatment efficacy evaluated at 1, 3, 6 and 12 months. Efficacy outcomes: international prostate symptom score (IPSS), quality-of-life (QoL) score, international index of erectile function-5 (IIEF-5), maximum urinary flow rate (Qmax), postvoid residual urine (PVRU) volume, and prostate volume (PV). Complications and sequelae also assessed. Comparisons performed with parametric/non-parametric tests.

**Results:**

Out of the 100 hundred patients, 84 qualified for the analysis (45 M-TURP/39 PK-TURP). No significant differences found in baseline characteristics or operative data, except for a longer operative time in PK-TURP (MD:7.9min; 95%CI:0.13-15.74; p=0.04). No differences found in IPSS, Qmax or PVRU volume. QoL score at 12 months was higher in PK-TURP (MD:0,9points; 95%CI:0.18-1.64; p=0.01). No differences in sexual function, PV, complications or sequelae were found. This study is “rigorous” (Jadadscale) and has a low risk of bias (Cochrane-Handbook).

**Conclusions:**

Based on this controlled trial, there is not significant variation in effectiveness and safety between M-TURP and PK-TURP for the treatment of BPH. The small difference in QoL between PK-TURP and M-TURP at the one-year follow-up is not perceivable by the patients and, therefore, not clinically relevant.

## INTRODUCTION

The field of minimally invasive surgical techniques for the treatment of lower urinary tract symptoms (LUTS) due to benign prostatic hyperplasia (BPH) (1) has experienced extraordinary technological development. A significant step forward is plasmakinetic transurethral resection of the prostate (PK-TURP).

Although PK-TURP procedures have a grade A recommendation in the guidelines (2), most studies comparing monopolar transurethral resection of the prostate (M-TURP) and PK-TURP are rated as “poor quality” on the Jadad scale (≤3 points) (3), has methodological robustness labeled as “low” according to the Cochrane Handbook checklist (4), or both. Therefore, despite extensive literature on TURP, high-quality data is needed to determine their relative effectiveness and the ideal patient profile for each technique, as M-TURP is still used in many centers in both developed and developing countries.

The objective of this study was to generate the much-needed high-quality data that meets the requirements of both the Jadad scale and Cochrane Handbook checklist and compare M-TURP and PK-TURP in terms of efficacy (primary outcome), quality of life (QoL), sexual function, intraoperative, perioperative, and complications as well as sequelae during the 12 months of follow-up (secondary outcomes).

## MATERIAL AND METHODS

### Patients

Men clinically diagnosed with LUTS in a tertiary-care public institution who required surgical treatment were invited to participate in the study from December 2014 to August 2016. Inclusion criteria were prostate volume (PV) of <80g on transrectal ultrasound (TRUS) with LUTS due to drug-refractory BPH or complications derived from BPH (acute urinary retention (AUR), recurrent hematuria, recurrent urinary tract infection (UTI) or bladder calculi), or both. Patients with a history of pelvic surgery or radiotherapy, neurogenic bladder dysfunction, or prostate carcinoma were excluded.

Randomized group assignment was ensured by using a table of random numbers. Only those patients who were willing to continue participating in the trial after surgery, had completed all the questionnaires during the follow-up, and did not present any malignancy requiring additional treatment, were eligible for inclusion in the analyses. An intention to treat analysis was conducted and, as usual for surgical trials, only patients were blinded to the procedure. Ethical approval of the institutional review board (IRB) (APR-14-72) was granted, and informed consent was obtained from all subjects.

### Study variables

Baseline characteristics included age, comorbidities, American Society of Anesthesiologists (ASA) classification, laboratory values including prostate-specific antigen (PSA), international prostate symptom score (IPSS), maximum urinary flow rate (Qmax), post voiding residual urine (PVRU) volume, PV by TRUS, QoL score, sexual activity, and international index of erectile function (IIEF-5). Direct questions were included to evaluate stress urinary incontinence (SUI), urge urinary incontinence with or without the need for drug use (UUIND and UUIWND), retrograde ejaculation, and dysuria. Drugs for LUTS and hemostasis used before surgery were recorded.

Operative outcomes included irrigation volume, operation time (from the first cut to catheter placement), changes in serum sodium and hemoglobin, amount of resected tissue, speed of resection (dividing resected tissue by operative time), length of stay, and length of the indwelling catheter.

Intraoperative, perioperative, and postoperative complications and sequelae at 1, 3, 6, and 12 months were recorded; when applied, complications were classified according to the Clavien-Dindo system (CDS). To measure bleeding, the variable hemorrhagic complications (HC) was created by grouping hematuria and clot retention. Efficacy outcomes (IPSS, QoL score, Qmax, PVRU volume, and PV by TRUS) and sexual function (sexual activity and IIEF-5 questionnaire) were recorded for the same periods.

Treatment failure was characterized by the need for a re-TURP (residual adenoma), readmission or reoperation, or by recurrent UTI.

### Surgical Technique

Patients were operated on by residents and senior urologists, as per the usual daily practice at our teaching institution. M-TURP was conducted with a 26-Ch Olympus/Storz resectoscope under continuous glycine irrigation (1.5% glycine, Baxter), using a monopolar stainless-steel loop connected to a ForceTriad™ generator (Medtronic) (cutting and coagulation, 120W and 80W). PK-TURP was performed with a 26-Ch Storz resectoscope under continuous irrigation with saline solution (0.9% NaCl, Baxter), using a bipolar Superloop platinum-iridium (Gyrus ACMI) resection loop connected to a Plasma Kinetic™ Superpulse generator (Gyrus-ACMI) (180W and 100W). The irrigation liquid was placed 2 meters above the ground in all cases, with the surgical table at 80cm from the floor. A Neptune 2® (Stryker) continuous aspiration system was used during surgery at 80mmHg. The Nesbit technique was performed in both groups and all procedures were performed under spinal anesthesia. Recovered tissue was collected and submitted for pathological exam. At the end of both procedures, a 22-Ch three-way Foley catheter was placed into the bladder with a closed drainage system. Continuous irrigation with saline solution was initiated at the end of the procedure and interrupted 24 hours after surgery; the irrigation was definitively withdrawn after 4 hours of clear urine (defined as being able to read the newspaper headline through the urine collection tube). Patients were discharged on the first postoperative day, and the catheter was removed during ambulatory care, 72 hours after surgery (if clear urine was observed). Blood tests were performed before discharge.

#### Statistical Analysis

This study was designed with an alpha error of 5% and a study power of 80% to detect differences of ≥3 points in the IPSS questionnaire records. Based on this and with the assumption of a 20% loss of patients during follow-up, the recommended initial sample size was 100 patients. Comparisons were conducted using the chi-square/Fisher test and T-Student/Mann-Whitney test as needed. Statistical significance was established at p <0.05 for all analyses. Statistical analysis was performed using IBM-SPSS v23.0 Statistics software.

## RESULTS

**Figure 1:**
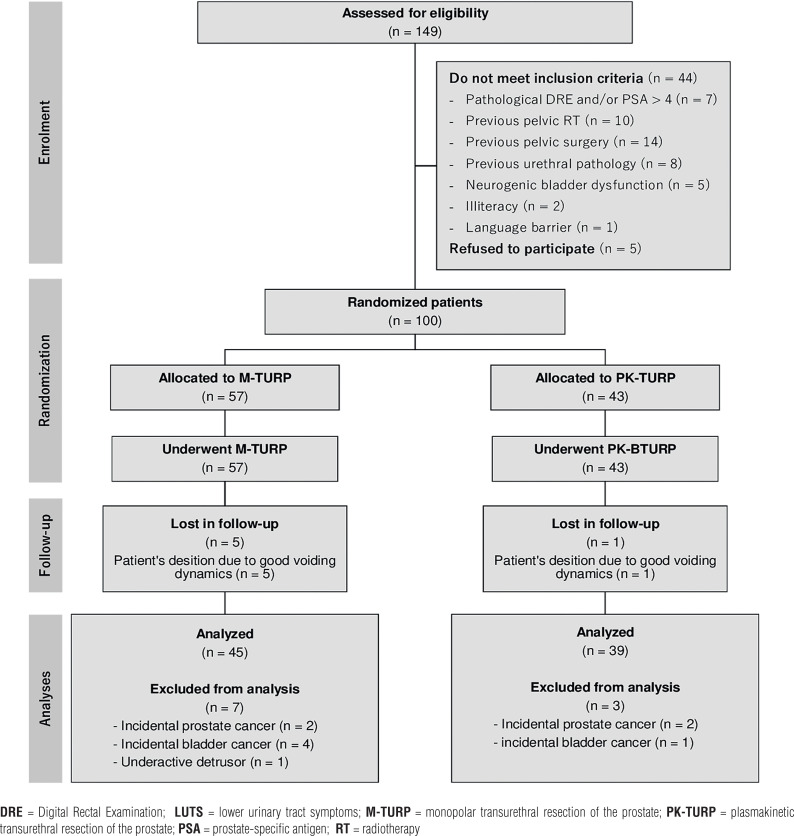
CONSORT diagram including randomization, treatment, and follow-up of subjects

Of the 100 randomized patients (53M-TURP and 47PK-TURP), 84 qualified for the analysis (45M-TURP and 39PK-TURP). Figure-1 summarizes the flow diagram.

There were no significant differences neither in baseline characteristics (Table-1) nor in operative data (Table-2) between the groups except for operative time (7.9 minutes longer for PK-TURP; 95%CI: 0.13-15.74; p=0.04). The histologic finding was BPH in all cases except for 3 cases with low-risk prostate cancer (7.7%), who were included in the active surveillance protocol.

**Table 1:** Baseline characteristics of eligible patients.

Parameters		M-TURP (n = 45)	PK-TURP (n = 39)	p value
		mean ± SD (range) / no. (%)	mean ± SD (range) / no. (%)	
Age, yr		64.9 ± 7.2 (51-82.7)	66.2 ± 7.1 (50.4-79.5)	0.41
BMI, Kg/m^2^		27.9 ± 4.2 (18.4-40)	26.8 ± 4.2 (21-41.5)	0.24
Hypertension		27 (60.0)	18 (46.2)	0.20
Diabetes mellitus		7 (15.6)	6 (15.4)	0.98
Smoker		7 (15.6)	6 (15.4)	0.98
ASA classification	ASA I	1 (2.3)	1 (2.6)	1.00
	ASA II	38 (84.4)	33 (84.6)	0.98
	ASA III	6 (13.3)	5 (12.8)	0.94
Serum PSA, ng/mL		2 ± 3 (0.3-13.7)	1.3 ± 0.9 (0.3-3.6)	0.37
Creatinine, mg/dL		0.9 ± 0.3 (0.6-3.3)	0.9 ± 0.1 (0.6-1.3)	0.20
eGFR, mL/min/1.73 m^2^		89.1 ± 23.6 (25-157.9)	89.2 ± 22.3 (59.6-154.1)	0.98
Serum sodium, mEq/L		140.8 ± 2.3 (134.6-146)	140.5 ± 2.5 (135-146.4)	0.54
Hemoglobin, g/dL		14.7 ± 1.3 (10.2-16.9)	15.1 ± 1.1 (12.8-17)	0.13
IPSS, points		24.7 ± 6.1 (11-34)	23.8 ± 6.2 (10-35)	0.50
QoL score, points		5.2 ± 0.8 (4-6)	4.8 ± 1.3 (0-6)	0.10
Sexual activity		37 (82.2)	28 (71.8)	0.25
IIEF score, points		11.7 ± 7.1 (1-25)	10.5 ± 7.9 (1-24)	0.47
Qmax, mL/s		10.9 ± 5.5 (4.5-33.2)	9.3 ± 4 (2.4-16.9)	0.16
PVRU volume, mL		60.6 ± 83.4 (0-360)	93.1 ± 91.1 (0-300)	0.12
PV by TRUS, mL		38.3 ± 17.4 (10-68)	41.4 ± 17.1 (19-69)	0.41
Stress urinary incontinence		0 (0.0)	0 (0.0)	—
UUIND		1 (2.2)	2 (5.1)	0.59
Retrograde ejaculation		5 (13.5)	3 (10.7)	0.52
Dysuria		5 (11.1)	6 (15.4)	0.56
UUIWND		13 (28.9)	9 (23.1)	0.54
Drugs for hemostasis	None	31 (68,9)	30 (76,9)	0,54
	Antiaggregants	12 (26,7)	5 (12,8)	0,11
	Anticoagulants	2 (4,4)	4 (10,3)	0,40
	None	1 (2.2)	1 (2.6)	1.00
Drugs for LUTS	Storage symptoms	1 (2.2)	2 (5.1)	0.59
	Voiding symptoms	43 (95.6)	36 (92.3)	0.65
	Drug failure	35 (77.8)	28 (71.8)	0.85
Indication of surgery	AUR	9 (20.0)	10 (25.6)	0.53
	Vesical calculi	1 (2.2)	1 (2.6)	1.00

**AUR** = acute urinary retention; **BMI** = body mass index; **eGFR** = estimated glomerular filtration rate; **IIEF** = International Index of Erectile Function; **IPSS** = International Prostate Symptom Score; **LUTS** = lower urinary tract symptoms; **M-TURP** = monopolar transurethral resection of the prostate; **PK-TURP** = plasmakinetic transurethral resection of the prostate; **PSA** = prostate-specific antigen; **PV** = prostate volume; **PVRU** = postvoid residual urine; **Qmax** = maximum urinary flow rate; **QoL** = quality of life; **SD** = standard deviation; **TRUS** = transrectal ultrasound; **UUIND** = urge urinary incontinence with need for drug use; **UUIWND** = urge urinary incontinence without the need for drug use.

**Table 2:** Operative data and intra/perioperative complications stratified by treatment

Parameters			M-TURP (n = 45) mean ± SD (range)/ no. (%)	PK-TURP (n = 39) mean ± SD (range)/ no. (%)	p value
**Operative data**					
Surgical experience		Resident	24 (53.3)	18 (46,2)	0.51
		Senior urologist	21 (46.7)	21 (53,8)	
Intraoperative irrigation volume, L			16±6.8 (3-36)	20.2±11.8 (6-60)	0.05
Operative time, min			39.7±14.1 (15-70)	47.7±21.4 (20-120)	**0.04**
Decrease in sodium, mEq/L			3.3±4.1 (-3-24)	2.2±3.6 (-5-18)	0.17
Decrease in hemoglobin, g/dL			0.9±1.1 (-1.2-4.6)	1±1.1 (-0.8-3.5)	0.53
Resected tissue weight, g			12.7±8.2 (1.9-34.7)	12.4±9.9 (2-37.3)	0.88
Resected tissue percentage, %			33.5±17 (6.3-82)	28.8±19 (7.4-79.4)	0.23
Speed resection, g/min			0.9±0.3 (0.3-1.7)	0.9±0.3 (0.3-1.5)	0.26
Hospital stay, d			1.1±0.4 (1-3)	1.1±0.3 (1-2)	0.95
Catheter duration, d			3.6±1.4 (3-11)	3.5±0.8 (3-7)	0.69
**Intraoperative complications**					
**TUR syndrome**			1 (2.2)	0 (0.0)	—
	0.9% NaCl and furosemide infusion		1 (2.2)	0 (0.0)	
**Blood transfusion**			0 (0.0)	0 (0.0)	—
**Perioperative complications**					
**Hematuria /clot retention**			8 (17.8)	7 (17.9)	0.99
	Conservative management (CDS I)		6 (13.3)	6 (15.3)	
	Surgical intervention (CDS III)		2 (4.4)	1 (2.5)	
**AUR after withdrawal of UC**			1 (2.2)	1 (2.6)	1.00
	Transient recatheterization (CDS I)		1 (2.2)	1 (2.5)	
**UTIWSS**			3 (6.7)	1 (2.6)	0.62
	Oral antibiotics (CDS I)		3 (6.6)	1 (2.5)	
**UTISS**			2 (4.4)	0 (0.0)	0.49
Intravenous antibiotics (CDS II)			2 (4.4)	0 (0.0)	

AUR = acute urine retention; CDS = Clavien-Dindo system; M-TURP = monopolar transurethral resection of the prostate; PK-TURP = plasmakinetic transurethral resection of the prostate; SD = standard deviation; TUR = transurethral resection of the prostate; UC = urethral catheter; UTISS = urinary tract infection with systemic symptoms; UTIWSS = urinary tract infection without systemic symptoms.

Perioperative complications were observed in 14 (31.1%) M-TURP and 9 (23.1%) PK-TURP patients (Table-2). Most cases were grade I, according to the CDS, with no significant differences between groups. No differences in bleeding complications were found. Early reoperation rates reached 4.4% and 2.6% for the M-RTUP and PK-RTUP groups, respectively, whereas readmission rates were 8.9% and 2.6%, respectively.

**Figure 2:**
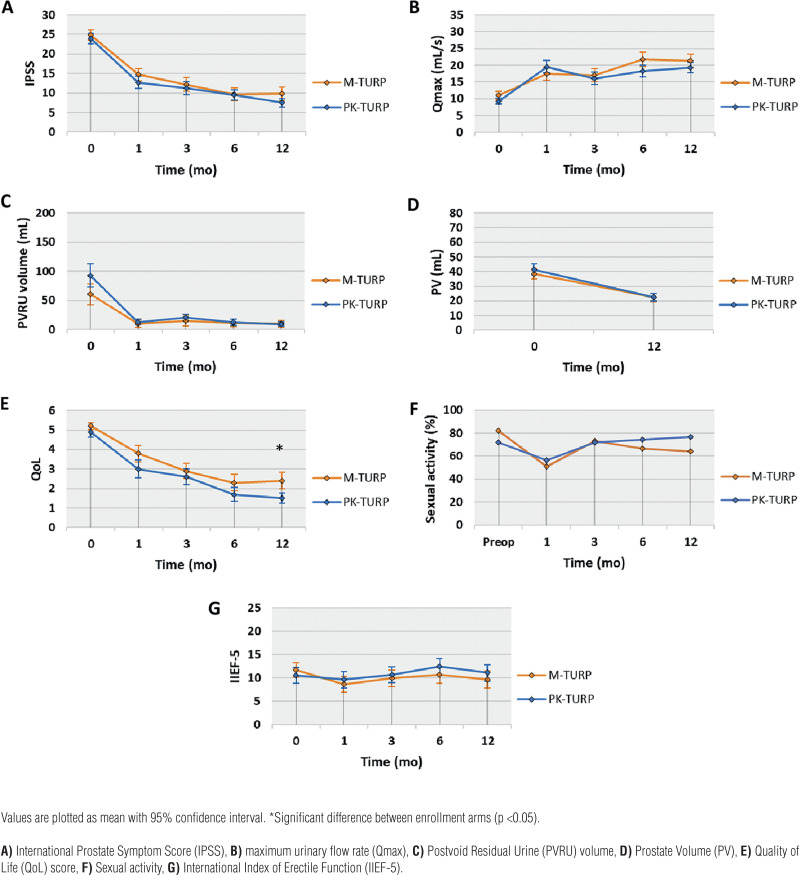
Outcome following treatment with M-TURP or PK-TURP

Treatment efficacy (IPSS, Qmax, PVRU volume, and PV) as the primary outcome, QoL, and sexual function at 1, 3, 6, and 12 months are listed in Table-3 and shown in Figure-2. IPSS, Qmax, PVRU volume and PV, and QoL showed significant improvement with both surgical techniques at each postoperative assessment when compared to baseline measurements. The only statistically significant difference between M-TURP and PK-TURP was the QoL score at 12 months (MD 0.9 points higher for PK-TURP; 95%CI: 0.18-1.64; p=0.01).

**Table 3:** Efficacy, quality of life, and sexual function stratified by treatment

Parameters		M-TURP		PK-TURP		p value
		Mean ± SD (range) / no. (%)	Mean change (%) / no. change (%)	Mean ± SD (range) / no. (%)	Mean change (%) / no. change (%)	
**IPSS, points**						
	1 mo	14.6 ± 7.4 (1-35)	-10.1 (-40.9)	12.7 ± 7.4 (3-34)	-11.1 (-46.6)	0.23
	3 mo	12.2 ± 8.2 (2-33)	-12.5 (-50.6)	11.3 ± 7.8 (2-34)	-12.5 (-52.5)	0.62
	6 mo	9.7 ± 7.7 (0-31)	-15 (-60.7)	9.4 ± 5.9 (2-28)	-14.4 (-60.5)	0.85
	12 mo	9.7 ± 8 (0-34)	-15 (-60.7)	7.4 ± 4.7 (1-18)	-16.4 (-68.9)	0.11
**Qmax, mL/s**						
	1 mo	17.5 ± 9 (5.7-45)	6.4 (58.2)	18.9 ± 8.3 (7.3-45.2)	10.3 (110.8)	0.45
	3 mo	18.2 ± 8.9 (3-41)	6.1 (55.5)	16.8 ± 9 (3.2-37.7)	6.8 (73.1)	0.47
	6 mo	21 ± 10.1 (4.4-45)	10.8 (98.2)	18.7 ± 8.1 (4.8-38)	9 (96.8)	0.24
	12 mo	21.2 ± 9.5 (3-46.7)	10.3 (93.6)	19.2 ± 7.2 (5.3-35.2)	10 (107.5)	0.27
**PVRU volume, mL**						
	1 mo	14.3 ± 28.8 (0-150)	- 50.5(-83.3)	13 ± 22.3 (0-100)	- 80.3(-86.2)	0.82
	3 mo	22.8 ± 44.9 (0-260)	-45.1 (-74.4)	18.2 ± 26.5 (0-100)	-72.3 (-77.6)	0.57
	6 mo	15.3 ± 28.9 (0-142)	-48.9 (-80.7)	14.7 ± 25 (0-130)	-80.4 (-86.3)	0.92
	12 mo	14 ± 28.3 (0-150)	-50.6 (-83.5)	8.3 ± 17.7 (0-70)	-83.6 (-89.7)	0.28
**PV by TRUS, mL**						
	12 mo	22.3 ± 13 (6-65)	-16.2 (-42.2)	22.5 ± 12.2 (6.5-61)	- 80.3(-86.2)	0.92
**QoL score, points**						
	1 mo	3.7 ± 1.9 (0-6)	-1.5 (-28.9)	2.9 ± 2.1 (0-6)	-1.9 (-39.6)	0.07
	3 mo	2.9 ± 1.8 (0-6)	-2.3 (-44.2)	2.6 ± 1.9 (0-6)	-2.2 (-45.8)	0.44
	6 mo	2.3 ± 1.9 (0-6)	-2.9 (-55.8)	1.6 ± 1.6 (0-9)	-3.2 (-66.7)	0.09
	12 mo	2.4 ± 1.9 (0-6)	-2.8 (-53.8)	1.5 ± 1.2 (0-4)	-3.3 (-68.7)	0.01
**Sexual activity**						
	1 mo	23 (51.1)	-14 (-31,1)	22 (56.4)	-6 (-15,4)	0.62
	3 mo	33 (73.3)	-4 (-8,9)	28 (71.8)	0 (0)	0.87
	6 mo	30 (66.7)	-7 (-15,5)	29 (74.4)	1 (2,6)	0.44
	12 mo	29 (64.4)	-8 (-17,8)	30 (76.9)	2 (5,1)	0.21
**IIEF-5, points**						
	1 mo	8.5 ± 7.9 (1-25)	-3.2 (-27.4)	9.5 ± 8 (1-25)	-1 (-9.5)	0.55
	3 mo	9.9 ± 8.2 (1-25)	-1.8 (-15.5)	10.7 ± 8 (1-25)	0.2 (1.9)	0.66
	6 mo	10.7 ± 8.5 (1-25)	-1 (-8.5)	12.4 ± 8.2 (1-25)	1.9 (18.1)	0.36
	12 mo	9.7 ± 8.6 (0-25)	-2 (-17.1)	11 ± 8.1 (0-25)	0.5 (4.8)	0.46

**IIEF** = International Index of Erectile Function, **IPSS** = International Prostate Symptom Score, **M-TURP** = monopolar transurethral resection of the prostate, **PK-TURP** = plasmakinetic transurethral resection of the prostate, **PSA** = prostate-specific antigen, **PVRU** = postvoid residual urine, **Qmax** = maximum urinary flow rate, **QoL** = quality of life, **SD** = standard deviation, **TRUS** = transrectal ultrasound.

Postoperative complications, sequelae, and treatment failures at 1, 3, 6, and 12 months are listed in Table-4. No significant differences between the groups were identified for these topics. Remarkably, all Clavien III complications, reoperations, and readmissions were restricted to three patients. Patients 45 and 60 deserve special mention since they presented the most complicated cases, whereas the third patient presented only one complication.

**Table 4:** Postoperative complications and sequelae stratified by treatment.

Parameters			Follow-up											
			1st month			3rd month			6th month			12th month		
			M-TURP (n = 45) no. (%)	PK-TURP (n = 39) no. (%)	p	M-TURP (n = 45) no. (%)	PK-TURP (n = 39) no. (%)	p	M-TURP (n = 45) no. (%)	PK-TURP (n = 39) no. (%)	p	M-TURP (n = 45) no. (%)	PK-TURP (n = 39) no. (%)	p
**Postoperative complications**														
	**Meatal stenosis**		2 (4.4)	2 (5.1)	1.00	5 (11.1)	2 (5.1)	0.44	2 (4.4)	0 (0.0)	0.49	4 (8.9)	2 (5.1)	0.68
		Meatus dilatation A (CDS II*)	2 (4.4)	2 (5.1)		5 (11.1)	2 (5.1)		2 (4.4)	0 (0.0)		4 (8.9)	2 (5.1)	
	**Urethral stricture**		0 (0.0)	0 (0.0)	—	2 (4.4)	1 (2.6)	0.99	2 (4.4)	2 (5.1)	1.00	1 (2.2)	2 (5.1)	0.59
		Urethral dilatation A (CDS II*)	0 (0.0)	0 (0.0)		1 (2.2)	1 (2.6)		1 (2.2)	2 (5.1)		0 (0.0)	2 (5.1)	
		Internal urethrotomy (CDS III*)	0 (0.0)	0 (0.0)		1 (2.2)	0 (0.0)		1 (2.2)	0 (0.0)		1 (2.2)	0 (0.0)	
	**Bladder neck contracture**		0 (0.0)	0 (0.0)	—	1 (2.2)	0 (0.0)	0.46	2 (4.4)	0 (0.0)	0.49	1 (2.2)	0 (0.0)	1.00
		Bladder neck incision (CDS III*)	0 (0.0)	0 (0.0)		1 (2.2)	0 (0.0)		2 (4.4)	0 (0.0)		1 (2.2)	0 (0.0)	
	**Stress urinary incontinence**		18 (40.0)	12 (30.8)	0.37	12 (26.7)	9 (23.1)	0.80	7 (15.6)	3 (7.7)	0.32	3 (6.7)	0 (0.0)	0.24
		Pelvic floor exercises (CDS I*)	18 (40.0)	12 (30.8)		12 (26.7)	9 (23.1)		7 (15.6)	3 (7.7)		3 (6.7)	0 (0.0)	
**UUIND**			9 (20.0)	3 (7.7)	0.10	3 (6.7)	3 (7.7)	0.99	3 (6.7)	2 (5.1)	0.99	0 (0.0)	0 (0.0)	—
	Drug use (CDS I*)		9 (20.0)	3 (7.7)		3 (6.7)	3 (7.7)		3 (6.7)	2 (5.1)		0 (0.0)	0 (0.0)	
	**UUIWND**		8 (17.8)	4 (10.3)	0.32	6 (13.3)	3 (7.7)	0.49	6 (13.3)	4 (10.3)	0.74	1 (2.2)	0 (0.0)	1.00
**Treatment failure**														
	Death related to TURP		0 (0.0)	0 (0.0)	—	0 (0.0)	0 (0.0)	—	0 (0.0)	0 (0.0)	—	0 (0.0)	0 (0.0)	—
	Recurrent UTI		0 (0.0)	0 (0.0)	—	0 (0.0)	0 (0.0)	—	0 (0.0)	0 (0.0)	—	0 (0.0)	0 (0.0)	—
	re-TURP		0 (0.0)	0 (0.0)	—	0 (0.0)	0 (0.0)	—	1 (2.2)	0 (0.0)	1.00	1 (2.2)	0 (0.0)	1.00
**Sequelae**														
	Retrograde ejaculation		17 (73.9)	13 (59.1)	0.29	24 (72.7)	18 (64.3)	0.47	21 (70.0)	17 (58.6)	0.36	23 (79.3)	18 (60.0)	0.15
	Dysuria		5 (11.1)	0 (0.0)	0.05	0 (0.0)	0 (0.0)	—	0 (0.0)	0 (0.0)	—	0 (0.0)	0 (0.0)	—

**CDS** = Clavien-Dindo system, **M-TURP** = monopolar transurethral resection of the prostate, **PK-TURP** = plasmakinetic transurethral resection of the prostate, **TURP** = transurethral resection of the prostate, **UTI** = urinary tract infection, **UTI** = urinary tract infection, **UUIND** = urge urinary incontinence with need for drug use, **UUIWND** = urge urinary incontinence without need for drug use.

A Dilatation in the office and self-dilatations

* The CDS only applies for complications <90 days.

Patient number 45 underwent M-TURP due to severe LUTS. The postoperative period was uneventful. However, one week afterward, urethral catheterization was needed due to AUR (Clavien I). He then was diagnosed with meatal stenosis (MS) and treated with self-dilatations (Clavien II). In the third month, he presented a new AUR due to a bulbar urethral stricture (US); it was treated with pneumatic dilatation at the office (Clavien II) in addition to self-dilatation. During his six-month follow-up visit, the urethroscopy revealed a normal urethral diameter. Nevertheless, a bladder neck contracture (BNC) and an obstructive residual adenoma had developed; the patient underwent therefore a bladder neck incision and re-TURP (Clavien IIIb). At his twelve-month follow-up visit, the urethroscopy showed a wide prostatic lodge and absence of US. Invasive urodynamic evaluation ruled out any bladder emptying obstruction and/or detrusor hyper/hypoactivity. Despite this, the patient continued with severe LUTS and a reduced QoL.

Patient number 60 underwent M-TURP because of AUR. The postoperative period was uneventful. Later, the patient experienced hematuria, requiring an endoscopic evaluation (Clavien IIIb) on the 20^th^ postoperative day. LUTS worsened, and Qmax deteriorated. Later, in the third month, he presented a bulbar US and BNC, requiring internal urethrotomy and a bladder neck incision (Clavien IIIb). After a brief subjective improvement, he developed drop-by-drop micturition because of recurrent US and BNC; surgical treatment was needed again (Clavien IIIb). At his twelve-month follow-up visit, the patient claimed to be experiencing severe LUTS. A new recurrence of US and BNC was confirmed, in addition to an obstructive residual adenoma. This time an internal urethrotomy, bladder neck incision, and re-TURP were performed. Further urodynamic evaluation ruled out bladder emptying obstruction and/or detrusor malfunction.

This study is “rigorous” according to the Jadad scale (achieving 4/5 points) and obtained a low risk of bias across selection, performance, detection, attrition, and reporting biases in accordance with the Cochrane Handbook checklist.

## DISCUSSION

Our study represents a unique contribution to previous literature due to the high methodological quality of its data. Based on our results, effectiveness and safety were not significantly different when comparing M-TURP and PK-TURP for BPH treatment. Regarding secondary outcomes, PK-TURP achieved a slightly superior QoL score than M-TURP after one year of follow-up, but it had a longer operative time.

As mention earlier, our study is rigorous according to the Jadad scale and has a low risk of bias in compliance with the Cochrane Handbook. By contrast, Mamoulakis et al. (4) have concluded that the methodological quality of previously published randomized controlled trials (RCTs) comparing M-TURP with PK-TURP is weak, negatively impacted for several reasons. First, the randomization method is only reported in a limited number of trials comparing M-TURP with PK-TURP (5-7). Secondly, only two studies (8, 9) have reported blinding for patients and researchers in a detailed manner. Additionally, CONSORT diagrams and figures for patient withdrawals are rarely reported and only a limited number of studies reported the acquisition of informed consent (7-9) or the approval of local IRB (7). Furthermore, the sample size calculation was only conducted in one RCT (9) and disclosure of sponsorship was appropriately mentioned in only one RCT (4). Finally, very few RCTs report the level of surgeon training. Because of all the reasons stated above, we are confident that our study represents a unique contribution to medical research, especially given that M-TURP is still used in many centers in both developed and developing countries.

In our study, both procedures were effective in treating LUTS secondary to BPH: at the one-year follow-up, PK-TURP and M-TURP provided the same functional results (IPSS, Qmax, PVRU, PV). When reviewing the literature, none of the previous RCTs reported significant differences between groups in IPSS at 12 months (6, 7, 10-14). Most studies did not show any difference in Qmax (6, 7, 9-13), whereas two studies found differences in favor of PK-TURP (MD 3.5mL/s; 95%CI:1.4-5.5 and MD 3.1mL/s; 95%CI:1.9-4.2) (15, 16). Interestingly, these differences are clinically relevant according to NICE guidelines, since their magnitude is ≥2mL/s (17). Regarding PVRU volume, some RCTs did not show any difference (10-12), whereas three reported a lower, clinically non-significant PVRU volume after PK-TURP (6, 7, 15).

According to our study, the QoL in PK-TURP was better at 12 months post-operation (MD 0.9 points; 95%CI: 0.18-1.64). In the literature, only Xie et al. (10) found a better QoL in favor of PK-TURP-B (MD 0.41 points; 95%CI:0.2-0.6), whereas the rest of the authors did not (6, 11-13). However, because these differences in QoL are <1 point, according to Dahm et al. (18), they are not perceivable by the patients and, therefore, they are not considered clinically relevant.

In our study, median PK-TURP surgical time was 20% longer than M-TURP (MD: 7.9 minutes; 95%CI: 0.13-15.74). However, this difference is probably not relevant in practical terms. Most of the previous RCTs comparing M-TURP and PK-TURP surgical time did not find significant differences (6-8, 10, 11, 13, 19, 20). Xie et al. (10) and Erturhan et al. (6) reported a shorter time for PK-TURP (MD 21 min; 95%CI:1.7-26.4 and MD 7.8 min; 95%CI:1.7-14.1, respectively). Surgical time is impacted by the technique, the size of the loop, and the surgeon's skills. In our experience, PK-TURP's longer surgical time was probably driven by: 1) the suggestion that slower resection speed is needed to achieve adequate cauterization in PK-TURP; 2) in M-TURP the surgeon tends to go as fast as possible due to the correlation between longer surgical time and TURP-syndrome. In our study, the longer surgical time of PK-TURP did not correlate with a higher amount of tissue resected.

Bleeding is a major issue since it may hinder the procedure and prolong the length of stay and the indwelling catheter. One of the potential advantages of bipolar devices is their higher hemostatic capacity resulting from their depth of coagulation (21). Additionally, the “cut-and-seal” effect of plasma may provide better cauterization and, therefore, reduce bleeding (22-24). However, and similar to previous reports (5, 8, 10-12, 15, 25), we did not find any significant difference in bleeding events between the procedures in our study. So far, only Erturhan et al. (6) showed a higher association of hemorrhagic phenomena with M-TURP (OR 0.1; 95%CI: 0.02-0.46).

Length of hospital stay and the indwelling catheter are the cornerstone of every surgical technique due to their direct relationship with health costs and health-related perceived QoL. In our study, the catheter was removed 72 hours after surgery if clear urine was observed. The average catheter duration for M-TURP and PK-TURP was not significantly different (3.6 vs. 3.5 days). Among existing RCTs, the drivers of catheter withdrawal are prostatic volume (9), urine color (6-9, 12, 13), or urine color after the first 24 hours (26, 27); the rest of RCTs do not specify the basis for catheter withdrawal decisions. The majority of studies have reported a shorter catheter duration in the PK-TURP group (1.5 vs. 2.5 days) (6, 9, 10, 12, 15, 16), whereas some found no significant differences (11, 13, 19). The average length of hospital stay in our study was 1.1 days for both techniques, which is remarkably sorter than what is reported by all existing RCTs. Three RTCs reported a shorter stay for the PK-TURP group (2.9 vs. 4.4 days) (6, 10, 16), whereas the rest found no significant differences (9, 12, 19). We believe that length of hospital stay and the indwelling catheter are probably more linked to the traditional routine at each institution rather than to the occurrence of complications, as in our case.

In our study, two-thirds of patients had sexual activity before surgery (82.2% vs. 71.8%). We observed a decrease in sexual activity after surgery despite not having prescribed sexual abstinence. While sexual activity tended to normalize, interestingly, after 12 months of follow-up it did not reach the baseline records with M-TURP (64.4%), whereas it exceeded them with PK-TURP (76.9%). Changes in erectile function rates did not accompany these changes in sexual, activity according to the IIEF-5 questionnaire. We did not find any argument to explain why two patients in the PK-TURP group developed de novo sexual activity after surgery. The tracking of sexual function after transurethral procedures is challenging and hardly reported in RCTs, which leads to insufficient data to obtain meta-analyzed results. The analysis of individuals studies reports that adverse sexual events following a PK-TURP procedure seemed comparable with those seen after M-TURP, remaining generally stable and with similar variations in each group (3).

In our study, we did not observe significant differences between M-TURP and PK-TURP in postoperative complications, sequelae, and treatment failure during the first year of follow-up. However, we had two patients with a very striking set of complications with M-TURP procedures. Both cases were operated by experienced urologists, the surgical time was shorter than average, and the length of stay and the indwelling catheter were standard. In addition, they did not show any striking or out-of-average baseline characteristics. From a statistical point of view, we cannot establish that M-TURP was the main cause of these patient's complications.

The participation of multiple surgeons with varying levels of surgical skills and experience could be considered a limitation of the study. However, our objective was to analyze the outcomes of both surgical techniques in the daily practice in a university institution. It should be noted that the sub-analysis based on level of surgical experience did not show statistically significant differences in either baseline characteristics or in primary and secondary outcomes. Due to the pathognomonic technical differences between M-TURP and PK-TURP, we were unable to blind the surgeons to the procedures being performed, which represents the main limitation of our study.

## CONCLUSIONS

Based on this controlled trial, which is considered high-quality data according to Jadad and Cochrane standards, there is no significant variation effectiveness and safety between M-TURP and PK-TURP for the treatment of BPH. The small difference in QoL between PK-TURP and M-TURP at a one-year follow-up is not perceivable by the patients and, therefore, not clinically relevant. Accordingly, M-TURP continues to be a valid option for the treatment of LUTS.

## Abbreviations

95%CI: confidence interval with 95% of probability
ASA: American society of anesthesiologists
AUR: acute urinary retention
BMI: body mass index
BNC: bladder neck contracture
BPH: benign prostatic hyperplasia
CDS: Clavien-Dindo system
eGFR: estimated glomerular filtration rate
HC: hemorrhagic complications
IIEF-5: international index of erectile function-5
IPSS: international prostate symptom score
IRB: institutional review board
LUTS: lower urinary tract symptoms
MD: mean difference
MS: meatal stenosis
M-TURP: monopolar transurethral resection of the prostate
OR: odds ratio
PK-TURP: plasmakinetic transurethral resection of the prostate
PSA: prostate-specific antigen
PV: prostate volume
PVRU: post voiding residual urine
Qmax: maximum urinary flow rate
QoL: quality-of-life
RCT: randomized controlled trial
SUI: stress urinary incontinence
TRUS: transrectal ultrasound
TUR: transurethral resection of the prostate
US: urethral stricture
UTI: urinary tract infection
UTISS: urinary tract infection with systemic symptoms
UTIWSS: urinary tract infection without systemic symptoms
UUIND: urge urinary incontinence with need for drug use
UUIWND: urge urinary incontinence without need for drug use
